# N7-methylguanosine methylation-related regulator genes as biological markers in predicting prognosis for melanoma

**DOI:** 10.1038/s41598-022-25698-x

**Published:** 2022-12-06

**Authors:** Jiehua Deng, Jiahua Lin, Chang Liu, Jiasong Li, Jun Cai, Xiyu Zhou, Xiong Li

**Affiliations:** 1 Department of Plastic and Aesthetic Surgery, The Second Affiliated Hospital of Guilin Medical University, No. 212 Renmin Road, Lingui District, Guilin, 541199 Guangxi Zhuang Autonomous Region China; 2 College of Otolaryngology Head and Neck Surgery, Chinese PLA General Hospital, Chinese PLA Medical School, 28 Fuxing Road, Beijing, 100853 China; 3 Department of Neurosurgery, The 924th Hospital of the Chinese People’s Liberation Army Joint Logistic Support Force, Guilin, 541002 Guangxi Zhuang Autonomous Region China

**Keywords:** Cancer genetics, Tumour biomarkers, Tumour immunology, Gene expression analysis, Software, Predictive markers, Prognostic markers

## Abstract

The aim of this study is to find those N7-methylguanosine (m7G) methylation-related regulator genes (m7GMRRGs) which were associated with melanoma prognosis and use them to develop a prognostic prediction model. Clinical information was retrieved online from The Cancer Gene Atlas (TCGA) and the Gene Expression Omnibus (GEO). R software was used to extract m^7^GMRRGs by differential expression analysis. To create a prognostic risk model, univariate and multivariate Cox regression analyses were employed for the evaluation of the prognostic significance of m^7^G methylation modifiers. Internal validation using cohort from TCGA (training set) and external validation using cohort from GEO (validation set) of the model were carried out. The model’s predictive performance was confirmed by using the Kaplan–Meier, univariate, and multivariate Cox regression, and receiver operating characteristic curve (ROC) by constructing column line plots incorporating clinical factor characteristics. Immune infiltration analyses were performed to assess the immune function of m^7^GMRRGs. Drug sensitivity analysis was conducted to study chemotherapeutic drug treatment cues. Prognostic models using four m^7^GMRRGs (*EIF4E3, LARP1, NCBP3,* and *IFIT5*) showed good prognostic power in training and validation sets. The area under the curve (AUC) at 1, 3, and 5 years for GEO-melanoma were 0.689, 0.704, and 0.726, respectively. The prediction model could distinctly classify patients with melanoma into different risk subgroups (*P* < 0.001 for TCGA-melanoma and *P* < 0.05 for GEO-melanoma). Clinical characteristics were taken into account in Cox regression and AUC analysis, which highlighted that the risk score served as an independent risk factor determining the prognosis of patients with melanoma. Immuno-infiltration analysis showed that m^7^GMRRGs could potentially regulate CD8^+^ T cells as well as regulatory T cells (Treg cells). Results of our study indicate a association between m^7^GMRRGs and melanoma prognosis, and the prognostic prediction model using m^7^GMRRGs may predict the prognosis of patients with melanoma well. Nevertheless, these results may provide a clue for potential better options of melanoma treatment but need further validation in futural studies.

## Introduction

Melanoma is regarded as one of the most aggressive cancers^1,2^. Individuals with melanoma usually have a poor prognosis since the cancer cells are highly invasive, migratory, and could metastasize at an early stage^3^. Although some progress has been made in recent years with targeted drug therapy and immunotherapy^4,5^, these treatments have not achieved significant success. Drug resistance is one of the factors that may stall good prognosis of melanoma patients^6^. Diagnosis in early stage of melanoma is essential as it may lead to a good prognosis with early treatments.

Previous studies have demonstrated that factors like tumor thickness, presence of anterior lymph node metastases, and presence of combined ulcers are strongly linked to the prognosis of individuals with melanoma^7,8^. However, only depending on clinical staging and histology to predict the prognosis of individual tumors has limited success^9,10^. The understanding of tumor biology at the molecular level has improved due to the advancement of high-throughput techniques which facilitates the identification of specific biomarkers and prognostic models. This may help more patients in early diagnosis and accept the corresponding treatment for better prognosis.

N7-methylguanosine (m^7^G) is the most prevalent internal modification that occurs in transfer RNA (tRNA) and non-coding RNA (ncRNA)^11^. m^7^G modifications usually occur at position 46 of the variable region, commonly occurring in fungi^12^, eukaryotes, archaea, and mammals^13^. It is crucial in regulating transcription and ribosomal RNA (rRNA) homeostasis. tRNA (m^7^G46) methyltransferase is shown to regulate m^7^G methylation. m^7^G46 forms a tertiary base pair with C13-G22 and stabilizes tRNA^14^. The regulators of m^7^G are linked to several pathological disorders and illnesses, according to numerous studies. m^7^G methyltransferase has been associated with the developing resistance to aminoglycoside antibiotics in Streptomyces tenebrarius^13^. In addition, m^7^G methyltransferase increases the infectivity of thermophilic bacteria by regulating the amount of tRNA modification^15^.

High throughput sequencing has revealed several RNA covalent modifications and provided therapeutic clues for cancers at the genetic and molecular levels. m^7^G is one of the most common covalent modifications. A recent study has shown that METTL1 methyltransferase mediates m^7^G within let-7e and promotes let-7e maturation, thereby exerting a tumor-suppressive effect on lung cancer^16^. Another study has demonstrated that m^7^G makes colorectal cancer cells more susceptible to cisplatin-based drugs by modulating the miR-149-3p/S100A4/p53 axis^17^. Increasing evidence has suggested a association between m^7^G and progression of mutiple cancers^18–20^. Nevertheless, there are few studies concerning relationship between m^7^G and prognosis in melanoma at present^21^.

Thus, multiple bioinformatics analyses were used on data collected from TCGA and GEO databases to investigate the possible association between m^7^G methylation-related regulator genes (m^7^GMRRGs) and melanoma prognosis and develop a prognostic prediction model using those genes for patients with melanoma, aiming to provide potential better options of melanoma treatment.

## Methods

### Data collection

The expression of RNA and clinical data of the melanoma patients was retrieved from the Cancer Genome Atlas (TCGA, https://tcga-data.nci.nih.gov/tcga/) and the Gene Expression Omnibus (GEO, https://www.ncbi.nlm.nih.gov/geo/) databases. TCGA database was utilized to develop the clinical prediction models, while the GEO database was employed for validation of the clinical prediction models. Clinical characteristics between the training and validation cohorts were collected and compared (Table 1).

**Table 1:** Comparison of clinical characteristics between the training and validation cohorts.

	Total (*n* = 597)	Training cohort (*n* = 447)	Validation cohort (*n* = 150)	*P* value
**Gender, n (%)**				0.243
Female	235 (39.4)	182 (40.7)	53 (35.3)	
Male	362 (60.6)	265 (59.3)	97 (64.7)	
**Age, n (%)**				0.428
≥ 60	211 (35.3)	162 (36.2)	49 (32.7)	
< 60	386 (64.7)	285 (63.8)	101 (67.3)	
**Stage, n (%)**				0.319
I	104 (17.4)	85 (19)	19 (12.7)	
II	209 (35)	152 (34)	57 (38)	
III	265 (44.4)	195 (43.6)	70 (46.7)	
IV	19 (3.2)	15 (3.4)	4 (2.6)	
**T, n (%)**				0.891
T0	38 (6.4)	30 (6.7)	8 (5.3)	
T1	78 (13)	56 (12.5)	22 (14.7)	
T2	124 (20.8)	92 (20.6)	32 (21.3)	
T3	149 (25)	110 (24.6)	39 (26)	
T4	208 (34.8)	159 (35.6)	49 (32.7)	
**M, n (%)**				0.109
M0	571 (95.6)	431 (96.4)	140 (93.3)	
M1	26 (4.4)	16 (3.6)	10 (6.7)	
**N, (%)**				0.907
N0	337 (56.4)	254 (56.8)	83 (55.3)	
N1	118 (19.8)	86 (19.2)	32 (21.4)	
N2	76 (12.7)	56 (12.5)	20 (13.3)	
N3	66 (11.1)	51 (11.5)	15 (10)	

*T* tumor stage, *N* lymph node, *M* distant metastasis.

### Identification of differentially expressed m^7^GMRRGs in melanoma

In total, 447 and 150 malignant tissues of melanoma from TCGA and GEO with their clinical information were acquired. From previous systematic reviews and the Molecular Signatures Database (MSigDB) database, a total of 29 m^7^GMRRGs (*METTL1, WDR4, NSUN2, DCP2, DCPS, NUDT10, NUDT11, NUDT16, NUDT3, NUDT4, NUDT4B, AGO2, CYFIP1, EIF4E, EIF4E1B, EIF4E2, EIF4E3, GEMIN5, LARP1, NCBP1, NCBP2, NCBP3, EIF3D, EIF4A1, EIF4G3, IFIT5, LSM1, NCBP2L, SNUPN)* were extracted^13,20^. The edgeR package was utilized to detect differentially expressed genes across melanoma and normal tissues.

### Functional enrichment analysis

The Kyoto Encyclopedia of Genes and Genomes (KEGG), Gene Ontology (GO), and the R package “clusterProfiler” were employed to study the biological role and signaling pathways linked to differentially expressed genes (DEGs).

### Development and validation of a prognostic signature model for m^7^GMRRGs in melanoma

We initially assessed the relationship between m^7^GMRRGs and overall survival (OS) using univariate Cox regression analysis. Prognosis-related moderators were those having a *P* value < 0.1 in univariate Cox regression analysis. Then, for the identification of key genes linked to prognosis and to create an optimum prognostic risk model, we employed stepwise multivariate Cox regression analysis.

Additionally, a risk score was derived using the following formula:

$${\text{Risk score}} = {\text{coef}}\left( {{\text{m}}^{{7}} {\text{GRNA1}}} \right) \times {\text{expr}}\left( {{\text{m}}^{{7}} {\text{GRNA1}}} \right){\text{coef}}\left( {{\text{m}}^{{7}} {\text{GRNA2}}} \right) \times {\text{expr}}\left( {{\text{m}}^{{7}} {\text{GRNA2}}} \right) \times ...{\text{ coef}}\left( {{\text{m}}^{{7}} {\text{GRNAn}}} \right) \times {\text{expr}}\left( {{\text{m}}^{{7}} {\text{GRNAn}}} \right).$$

where coef (m^7^GRNAn) was the weak effect associated with survival and expr (m^7^GRNAn) was the weak effect of expression.

Using the median risk scores, melanoma patients were sorted into a high- and a low-risk subtype subgroup. The survival R package was used to do a Kaplan–Meier analysis for comparing the survival rates between the two subgroups. ROC curves were created to test the performance and accuracy of the prognostic prediction model.

### Construction of the nomogram

Column line graphs were constructed based on several clinical factors, including risk score, gender, age, and TNM staging, using the R package rms software. The column line graphs predicted the survival rates of individuals with melanoma at 1, 3, and 5 years. Calibration curves for the corresponding column line graphs were plotted using R-pack survival rates to test the predictive power of the column line graphs.

### Immunological analysis

The R package "CIBERSOFT" algorithm was employed to study the tumor-infiltrating immune cell composition or cellular immune response across the high- and low-risk subgroups for melanoma in accordance with m^7^GMRRGs. The R package was utilized to visualize the findings. We used single-sample set gene enrichment analysis (ssGSEA) to analyze tumor-infiltrating immune cell subpopulations and immune function among both subgroups. In addition, we analyzed the potential immune checkpoints using previous literature.

### Drug sensitivity analysis

The Wilcoxon signed-rank test was performed using the R package "pRRophetic". "ggplot2" was utilized for studying the association of risk scores with sensitivity for chemotherapeutic agents commonly used in melanoma. *P* < 0.05 was considered as significant.

### Data analysis

Statistical analysis was carried out with R software(Version3.6.3).All of the R packages listed before were downloaded from http://www.bioconductor.org. Samples with incomplete or missing data were excluded. The expression profiles of 29 genes in melanoma and normal tissues from the TCGA database were compared using univariate analysis and chi-square tests to study the link between clinical features and m^7^GMRRGs. The survival differences between two subgroups with various levels of m^7^G RNA expression were compared using Kaplan–Meier curves. The Wilcoxon test assessed the differences between clinical factors and risk scores between subtypes. *P* < 0.05 was taken as a criterion of a significant difference.

## Results

### Expression profiles of m^7^GMRRGs in melanoma

**Figure 1:**
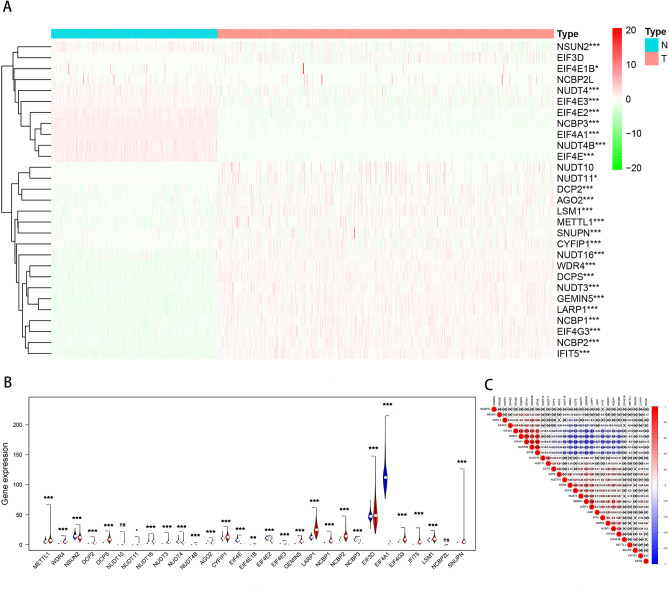
The profiling of m7GMRRGs in melanoma. (**A**) Heat map of 29 m^7^GMRRGs in tumor and normal tissues (upregulated are marked in red, downregulated are marked in green; *P < 0.05, *P < 0.01, and ***P < 0.001). (**B**) Vioplot visualization of m^7^G RNA methylation regulators in melanoma (red is melanoma, blue is normal tissue). (**C**) Spearman correlation analysis of 29 m^7^GMRRGs in melanoma. m^7^G*MRRGs* N7-methylguanosine methylation-related regulator genes.

We plotted heat maps and violin plots to understand RNA expression levels of m^7^GMRRGs between 447 cases of melanoma and 233 cases of normal tissues (Fig. 1A,B) from TCGA. Red and green subscales shown in Fig. 1A signify relatively high or low RNA expression levels, correspondingly. The m^7^GMRRGs showed significantly higher RNA expression levels in melanoma when compared with normal tissues were *DCP2*, *AGO2*, *LSM1*, *METTL1*, *SNUPN*, *CYFIP1*, *NUDT16*, *WDR4*, DCPS, *NUDT3*, *GEMIN5*, *LARP1*, *NCBP1*, *EIF4G3*, *NCBP2*, *IFIT5* (all *P* < 0.001). The RNA expression level of *EIF3D* and *NUDT10* did not show significant differences between groups (*P* > 0.05). The association of the 29 m^7^GMRRGs with each other were shown in Fig. 1C using Pearson correlation analysis.

### Enrichment analysis

**Figure 2:**
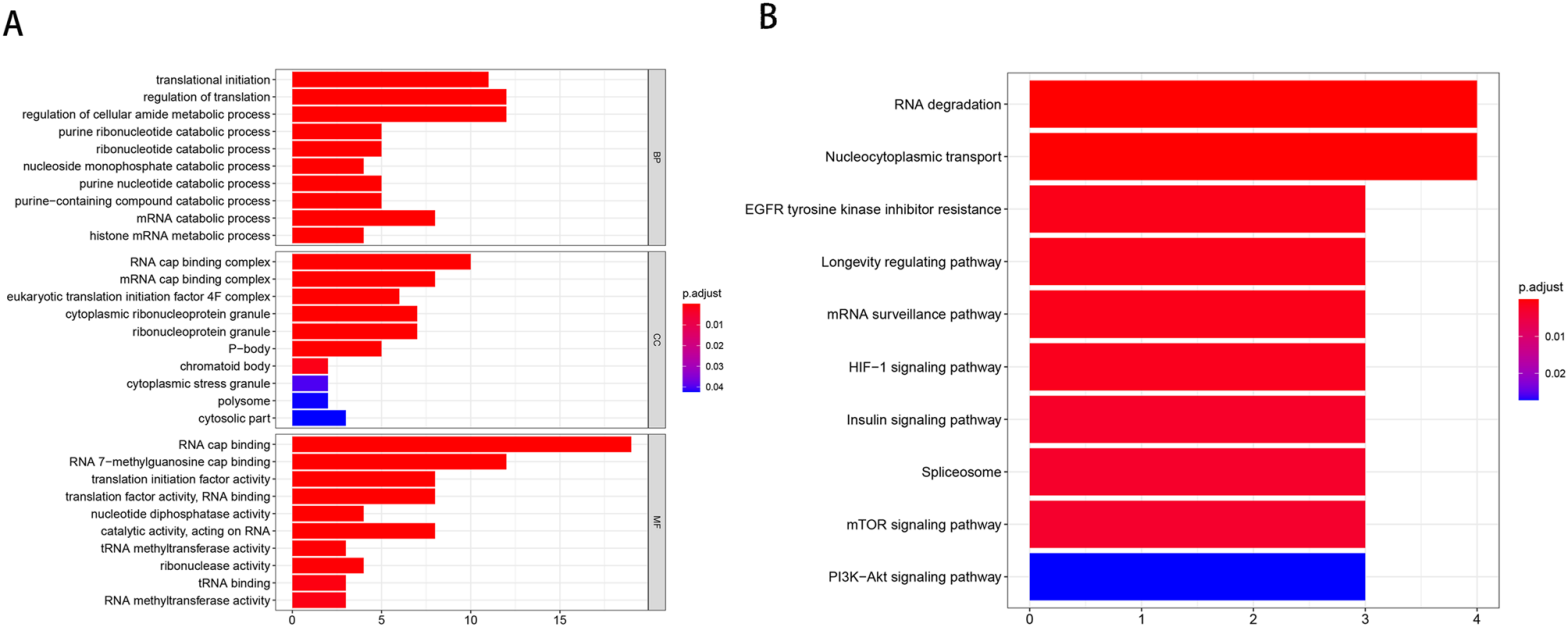
Analysis of DEGs based on GO (**A**) and KEGG (**B**). The x-axis indicates the number of m^7^GMRRGs enriched on each GO and KEGG. Rectangular colors indicate the significance of enrichment. *GO* gene ontology, *KEGG* Kyoto Encyclopedia of Genes and Genomes, *DE*-*m*^*7*^*G* differentially expressed m^7^GMRRGs, *BP* biological process, *CC* cellular component, *MF* molecular function, *DEGs* differentially expressed genes, *m*^*7*^*GMRRGs* N7-methylguanosine methylation-related regulator genes.

For further exploring the possible signaling pathways, biological roles, and functional analysis involved in m^7^GMRRGs, enrichment analysis was carried out employing GO and the KEGG. GO test identified biological pathways in which m^7^GMRRGs were involved were translation initiation and regulation, regulation of cell amide metabolism, RNA cap binding, and pathways related to the biological function of RNA 7-methylguanosine cap binding (Fig. 2A). KEGG enrichment analysis showed that m^7^GMRRGs also play a role in the RNA degradation, nucleocytoplasmic transport, epidermal growth factor receptor regulation, tyrosine kinase inhibitor resistance, longevity regulation pathway, mRNA monitoring, and insulin signaling pathway (Fig. 2B).

### Identification of a prognostic prediction model using m^7^GMRRGs in melanoma

**Figure 3:**
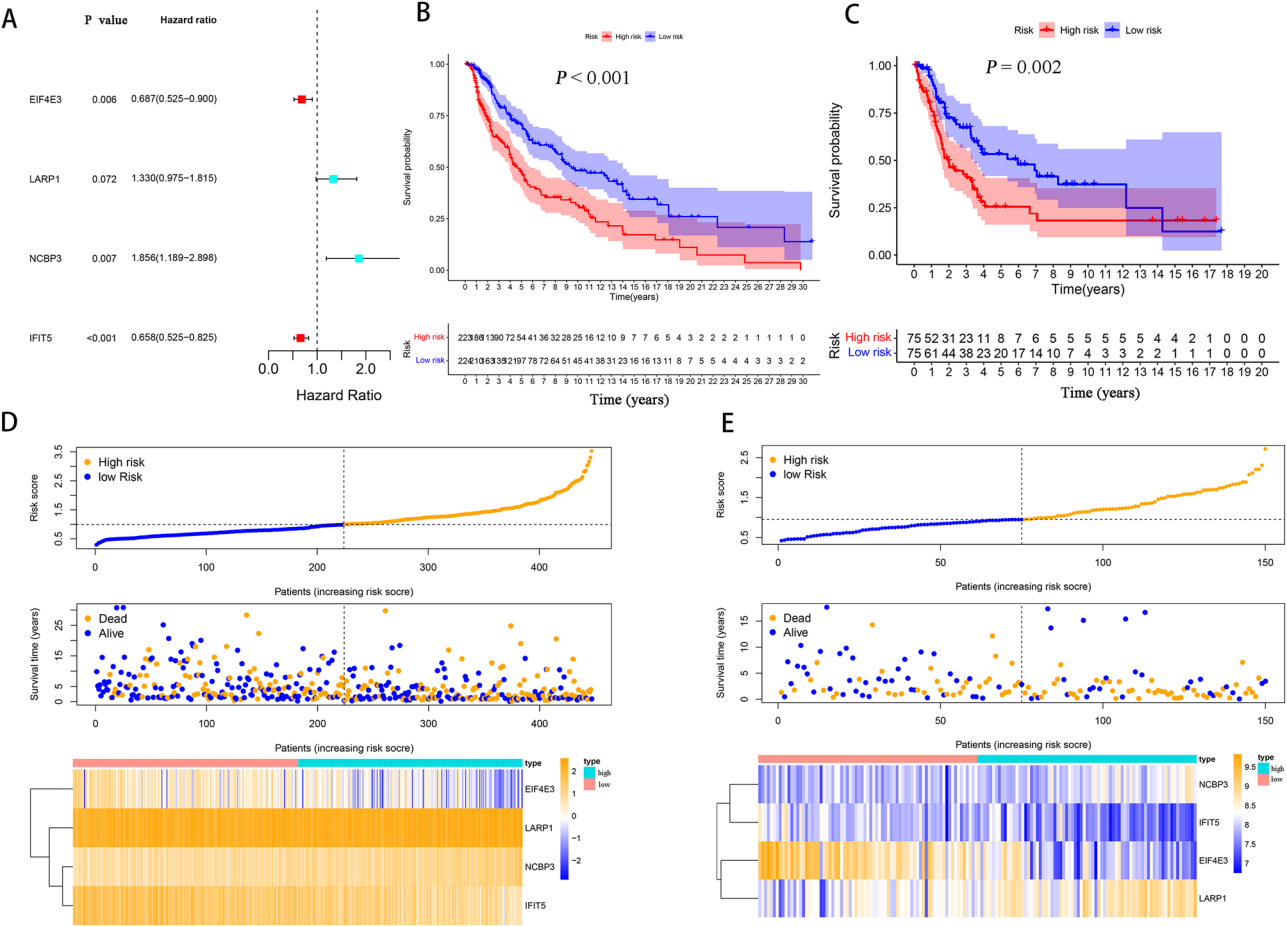
Construction of an prognostic prediction model for melanoma patients. (**A**) HR and *P* values of the four m^7^GMRRGs in the prognostic model. (**B**) Survival curves of patients in the high- and low-risk subgroups of the training set. (**C**) Validation of survival curves for patients in the pooled high-risk and low-risk subgroups. (**D**) Distribution of risk scores, survival status, and RNA expression of the four m^7^G regulator genes between the high-risk and low-risk subgroups in the training set. The top panel shows patients' risk scores. The middle panel depicts patients' survival status and survival time distributed by risk score. The bottom panel shows the braided hotspots for the four predictors by risk score. (**E**) Distribution of risk scores, survival status, and RNA expression of the four m^7^G regulator genes between the high-risk and low-risk subgroups in the validation set. *m*^*7*^*GMRRGs* N7-methylguanosine (m^7^G) methylation-related regulator genes.

Using Kaplan–Meier analysis for prognosis on the basis of the whole TCGA dataset with 447 cases, the prognostic significance of differentially expressed m^7^GMRRGs in melanoma patients was investigated. m^7^GMRRGs were tested in multivariate Cox regression and stepwise regression after having a *P* < 0.1 in Kaplan–Meier^22,23^. Then four key genes (*EIF4E3, LARP1, NCBP3, IFIT5*) were finally selected to create a prognostic prediction model of m^7^GMRRGs characteristics. Hazard ratio (HR) and *P* values for the four genes are shown in Fig. 3A.

As per the median risk score, the patients were divided into high- and low-risk subtypes. The survival study revealed that the prognostic prediction model demonstrated a remarkable capacity to distinguish between good and poor outcomes in patients with melanoma in the training set. Patients with the low-risk subtype survived considerably better in comparison with those having the high-risk subtype (*P* < 0.001; Fig. 3B). The same model was used to calculate each patient’s risk score and study the relation of risk score with survival status in the training set. The validation set on the basis of the whole GEO dataset with 150 cases was used to further validate the prognostic prediction model. Patients in the high-risk subgroup had substantially shorter survival in comparison with patients in the low-risk subgroup in the validation set (*P* = 0.0026; Fig. 3C). Figure 3D,E depict how risk scores, survival status, and RNA expression of the four m^7^GMRRGs were distributed between the high-risk and low-risk subgroups in the training and validation sets.

**Figure 4:**
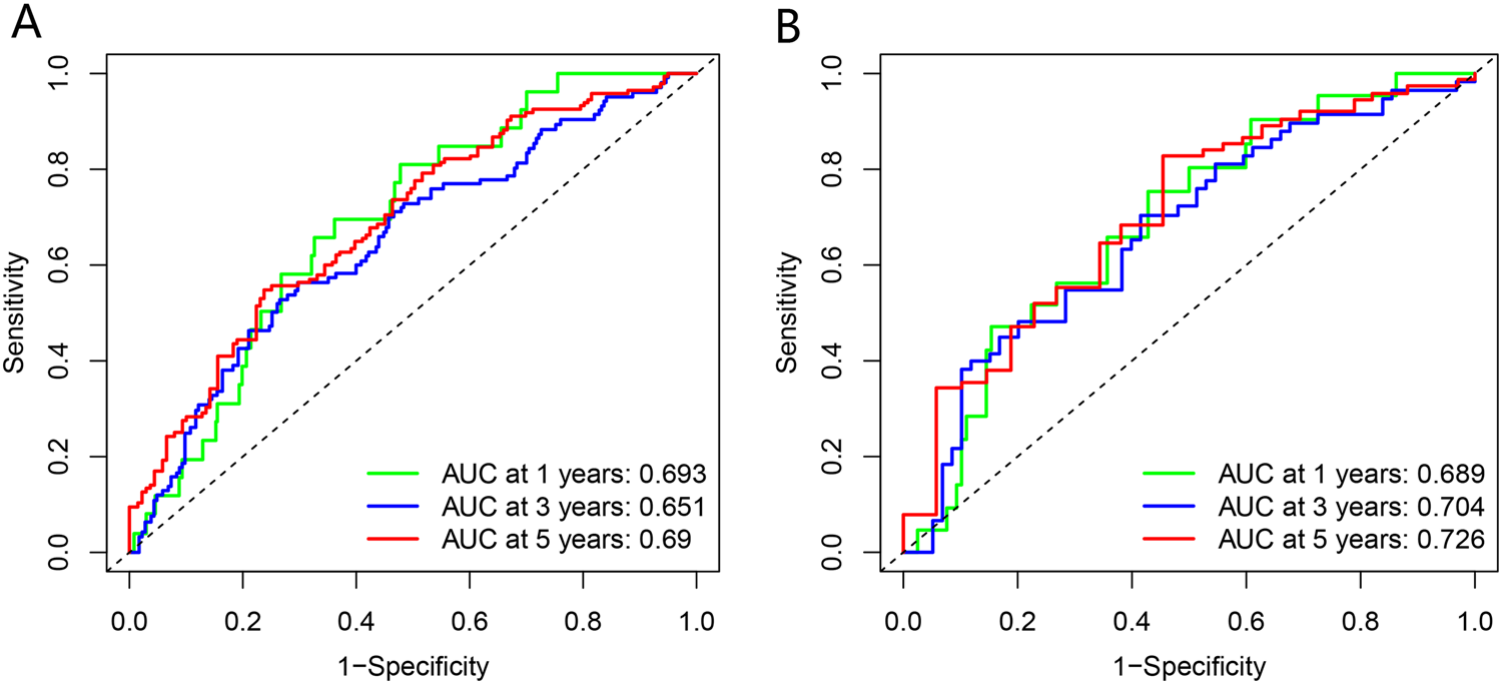
Validation of the prognostic, predictive power of four selected m^7^GMRRGs. (**A**) ROC curves of prognostic prediction models in the training set. (**B**) Validation of the ROC curves of the pooled prognostic prediction model. *m*^*7*^*GMRRGs* N7-methylguanosine methylation-related regulator genes.

ROC curves were generated to study the accuracy of the prognostic prediction model. Figure 4A shows the 1, 3, and 5-year ROC curves’ AUC values for the m^7^G features in the training set, which were 0.693, 0.651, and 0.69, respectively, whereas, Fig. 4B shows the prognostic model as 0.689, 0.704, and 0.726, respectively. Together, these findings show the accuracy of the four m^7^GMRRGs screened for prognostic prediction models for melanoma.

### Correlation of the prognostic prediction model and clinical factors in melanoma

**Figure 5:**
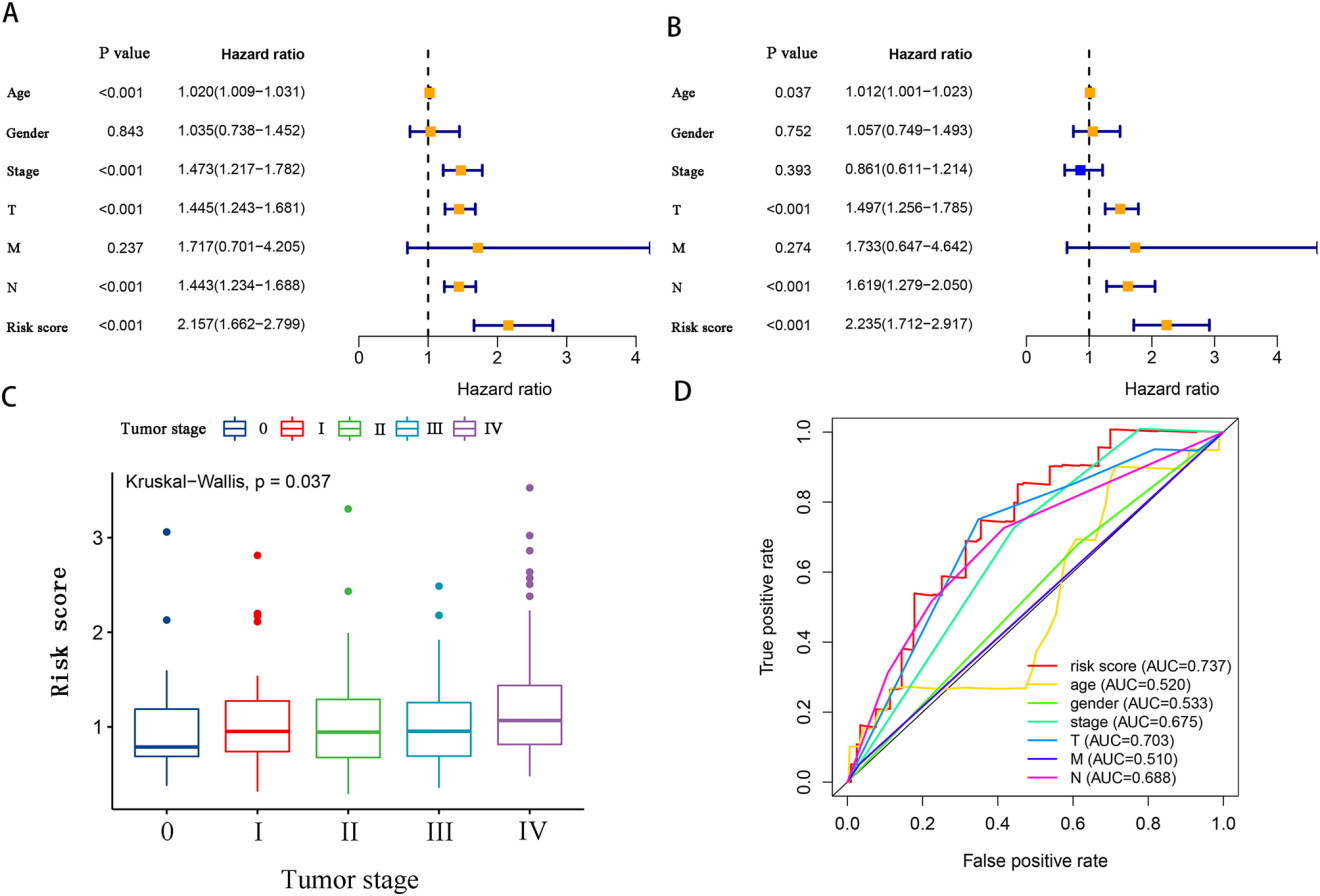
Relationship between prognostic prediction models and clinical factors in melanoma patients. (**A**) Univariate Cox analysis of clinical features based on the training cohort. (**B**) Multivariate Cox analysis of clinical features based on the training cohort. (**C**) Association between risk scores and clinical features of patients with melanoma. (**D**) ROC curves for risk scores and clinical factors. Clinical factors include age, gender, tumor stage, N (lymph node metastasis), T (tumor size), and M (distant metastasis).

To evaluate the correlation of the prognostic prediction model with clinical factors in patients with melanoma, the differences in risk scores between age, gender, tumor stage and size, lymph node, and distant metastasis were analyzed using the Wilcoxon test on the basis of the whole TCGA and GEO datasets with 597 cases. As shown in Fig. 5C, the risk score was considerably to be associated with tumor size (*P* = 0.037). No significant differences were observed in age, gender, tumor stage, lymph node, and distant metastasis (Supplementary Fig. 1). In addition, Kaplan–Meier analysis revealed a substantial association between age (HR = 1.020), tumor stage (HR = 1.473), tumor size (HR = 1.445), lymph node metastasis (HR = 1.443), and risk score (HR = 2.157; all *P* < 0.001) with poor prognosis (Fig. 5A). Multivariate Cox regression revealed that age (HR = 1.012, *P* = 0.037), tumor size (HR = 1.497, *P* < 0.001), lymph node metastasis (HR = 1.619, *P* < 0.001), and risk score (HR = 2.235, *P* < 0.001) were substantially independently associated with poor prognosis (Fig. 5B).

ROC curves were utilized to study the sensitivity and specificity of the risk score in predicting prognosis with all the 597 cases. The 5-year ROC curves’ AUC values of the risk score was 0.737, which was greater compared with other clinical factors, indicative of the reliability of the prognostic prediction model on the basis of the four m^7^GMRRGs (Fig. 5D).

**Figure 6:**
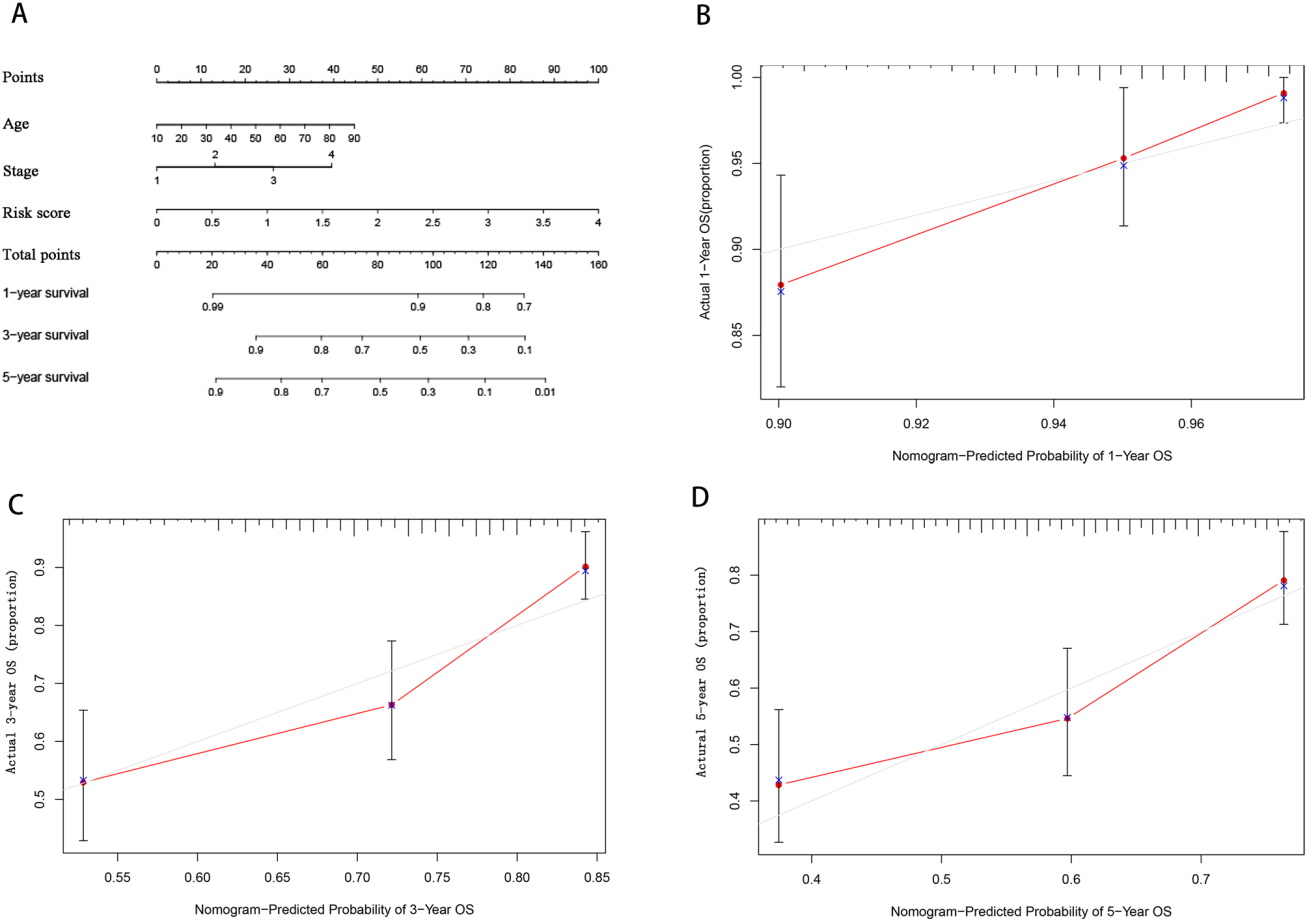
Nomogram development and evaluation. (**A**) a nomogram on the basis of age, stage, and risk score predicts melanoma patients’ prognosis. (**B–D**) Calibration curves for predicting overall survival in melanoma patients at 1 year (**B**), 3 years (**C**), and 5 years (**D**).

The three independent prognostic factors, age, tumor stage, and risk score, were combined to predict melanoma patients' 1, 3, and 5-year survival rates by plotting column line plots (Fig. 6A). To test the calibration of the column line plots, the predicted and the actual 1, 3, and 5-year survival rates were compared. The results presented good agreement between the predicted and actual survival rates of the calibration curves (Fig. 6B–D).

### Correlation of prognostic prediction models and immunoassays

**Figure 7:**
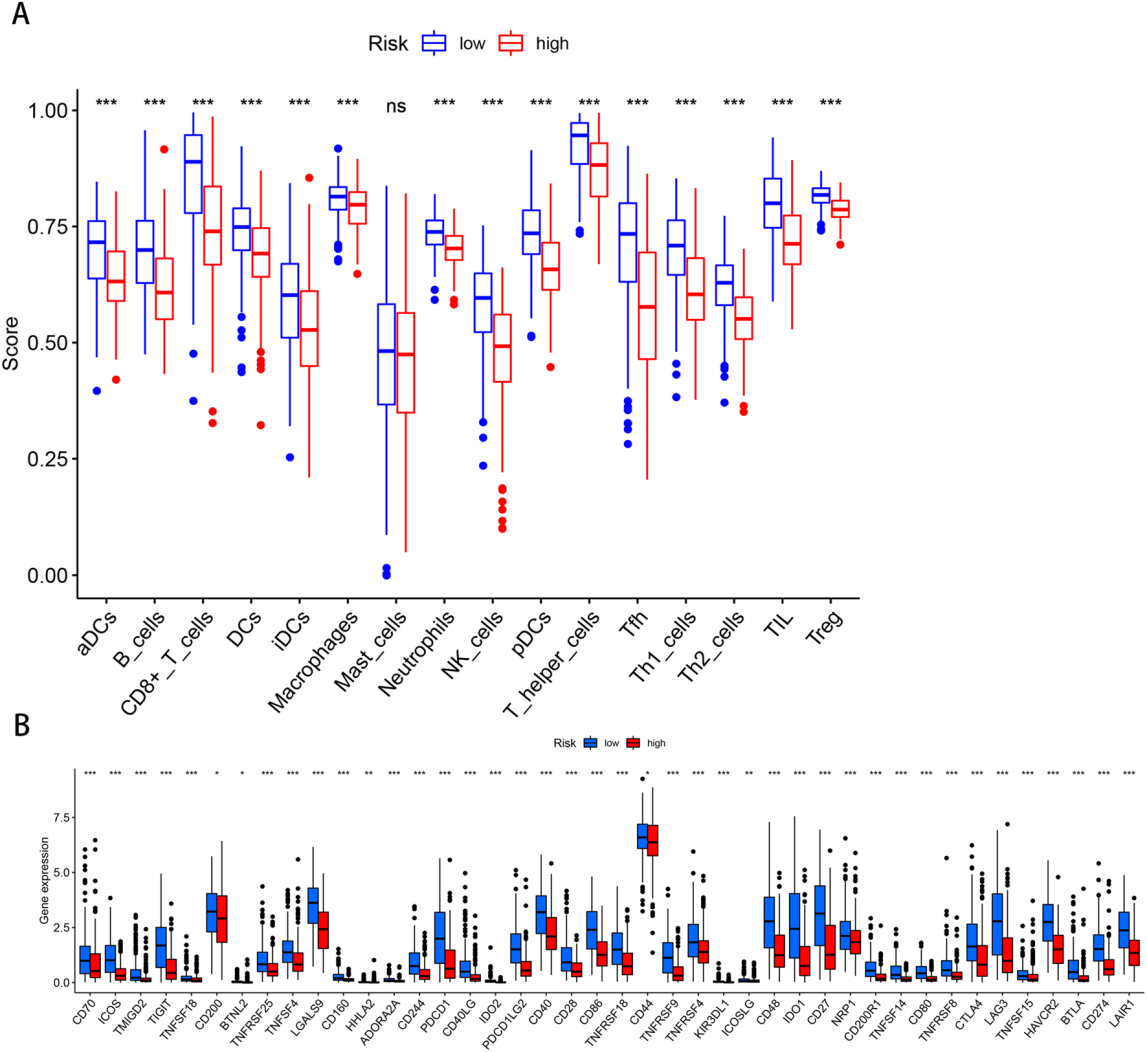
Correlation analysis of prognostic prediction models with immunological and chemotherapeutic drug sensitivity. (**A**) Correlation analysis based on risk score of subgroups with immune cell subpopulations and related functions. (**B**) Expression of immune check loci in high-risk and low-risk subgroups of melanoma.

The CIBERSOFT algorithm and ssGSEA were used to compare the cellular composition and cellular immune responses between the high- and low-risk subgroups distinguished by the prognostic prediction model. The high-risk subgroup had significantly lower infiltration of B cells, CD8^+^ T cells, dendritic cells, neutrophils, NK cells, macrophages, mast cells, Plasmacytoid dendritic cells, T helper cells, T follicular helper cells, Th_1_ cells, Th_2_ cells, tumor-infiltrating lymphocytes, and regulatory T cells than that of the low-risk subgroup (*P* < 0.001; Fig. 7A). The difference in immune check site expression between both subgroups was explored for their potential role in immunotherapy. Remarkable variations were observed in the expression of a variety of immune checkpoint loci between subgroups, most notably in CD70*,* TIGIT, PDCD1 (PD-1), LAG3, and LGALS9 (*P* < 0.001; Fig. 7B).

**Figure 8:**
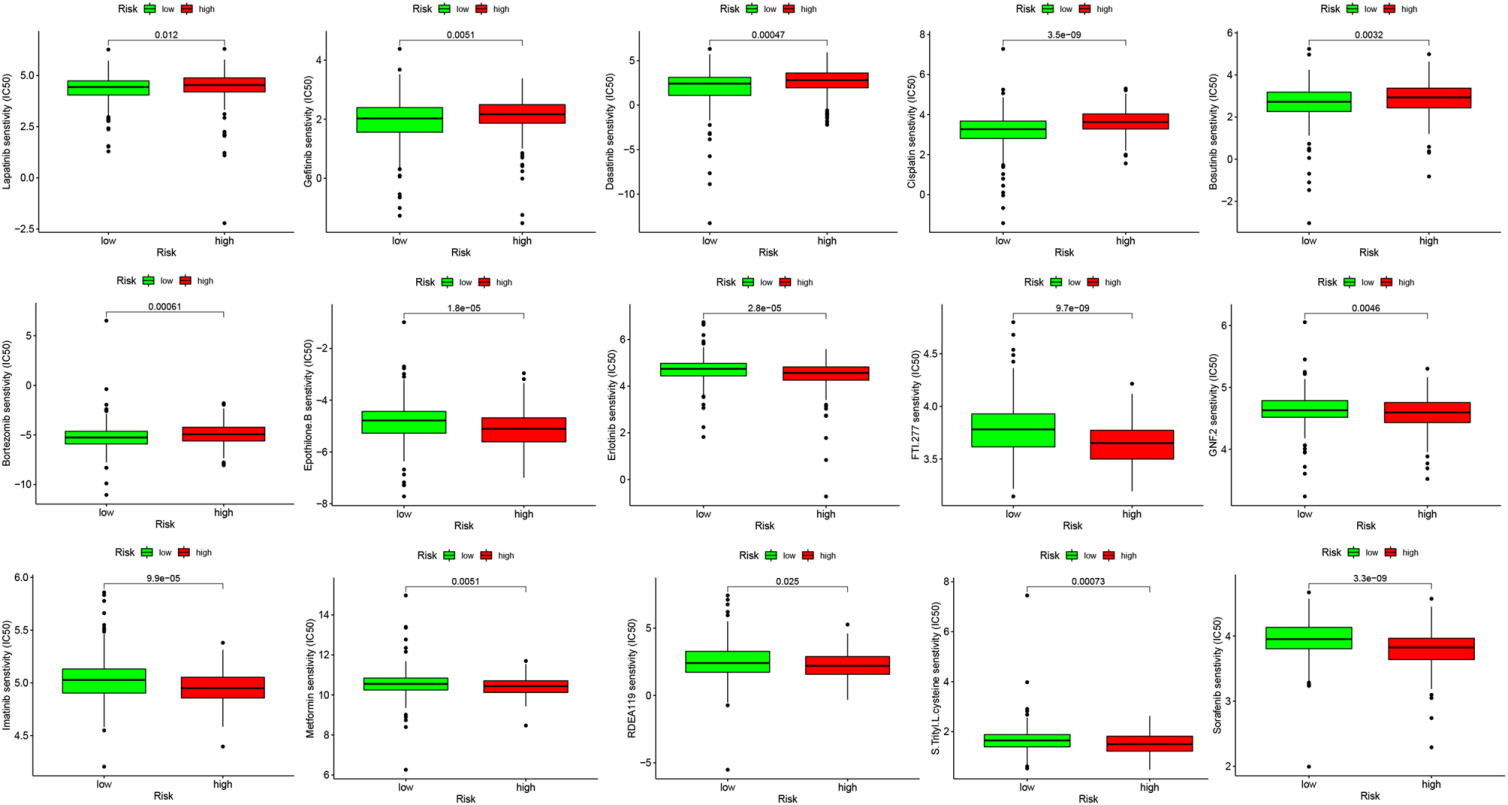
Correlation analysis of melanoma risk score and chemotherapy drug sensitivity.

A correlation analysis was also conducted between risk scores and sensitivity to common chemotherapeutic agents. As illustrated in Fig. 8, patients in the high-risk subgroup showed higher sensitivity to chemotherapeutic agents such as Bortezomib, Bosutinib, Cisplatin, Dasatinib, Gefitinib, and Lapatinib; while the patients in the low-risk subgroup showed higher sensitivity to Epothilone B, Erlotinib, FTI.277, GNF.2, Imatinib, Metformin, RDEA119, S-Trityl-L-cysteine, Sorafenib, and other common chemotherapeutic agents as well as emerging drugs (P < 0.05).

## Discussion

Melanoma is one of the most widely known types of cancer around the globe, with a constant increase in cases each year^24^. Approximately 300,000 new cases of melanoma are added each year. Based on incidences and mortality rates calculated in 2020, based on the estimate, it is believed that by 2040, 510,000 new melanoma cases will be added. Therefore, melanoma control prevention and treatment continue to pose a significant challenge globally^25^. Melanoma most commonly develops on the skin but may also develop in the uvea or mucosa^26^. Cutaneous melanoma is the most aggressive form of melanomas, with early metastasis, high mortality^27^, and a 5-year survival rate is < 20% for patients with advanced melanoma^28^.

Early diagnosis and treatment of melanoma are crucial in improving prognosis and survival. However, classification based on clinical features has low accuracy in prognosis, diagnosis, and treatment of melanomas due to individual differences amongst the patients^29^. Therefore, to optimize the performance of the prediction model, it is essential to understand the molecular features underlying melanoma formation and metastasis. Previous studies have shown epitranscriptomics as a key player in cancer development and progression, making it a valuable candidate for diagnosis, prognosis, and therapeutics^30–32^.

Methylation involves the transfer of methyl group onto nucleotide by catalysis. Methylation is one of the essential chemical modifications of nucleic acids, which regulates gene expression and is associated with many diseases, such as cancer and neurodegenerative diseases e.g., Alzheimer’s disease^33^. RNA methylation modifications are among the most common epigenetic modifications. m^7^G formation results from the addition of methyl groups to the N7 position of guanosine^13^. RNA guanine-7 methyltransferase modifies mRNA by cap addition, which regulates mRNA responses and gene expression^34^. In recent studies, RNA methylation has been shown to be linked to tumor immunity^35^. *WDR4* is crucially involved in promoting the proliferation of hepatocellular carcinoma by mediating m^7^G methylation^36^. Moreover, aberrant m^7^G methylation is closely linked to ischemic disorders^37^, soft tissue sarcoma^38^, lung cancer^39,40^, and gastrointestinal cancer^41^. Nevertheless, there is few studies concerning relationship between m^7^G and prognosis in melanoma at present. Their association needs to be studied in detail.

First, a comparison of RNA expression levels of m^7^GMRRGs in melanoma and normal tissues was performed based on the data retrieved from the TCGA database. The outcomes revealed that the RNA expression level of m^7^GMRRGs was considerably elevated in melanoma in comparison with their RNA expression level in normal tissues. Enrichment analysis revealed that the m^7^GMRRGs are involved in pathways regulating pathways associated with innate and adaptive immune cell expression and metabolic pathways. Previous studies have suggested activating mutations in tyrosine kinase regions as proto-oncogenes. Tyrosine kinase inhibitors exert anti-tumor effects by inhibiting the expression of proto-oncogenes^42^. In addition, insulin can activate proto-oncogenes via PI3K signaling via an intracellular pathway^43^. Enrichment analysis reveals pathways like tyrosine kinase inhibitor and insulin signaling are regulated by m^7^GMRRGs. These findings suggests the possible role of RNA expression level of m^7^GMRRGs expression in targeted therapeutics in melanoma.

To understand the role of m^7^GMRRGs in melanoma prognosis in further detail, we selected four m^7^GMRRGs (EIF4E3, LARP1, NCBP3, and IFIT5) to develop prognosis prediction model. The melanoma patients were sorted into high- and low-risk subgroups according to the risk score results derived using univariate and multivariate Cox analyses. A poor prognosis was observed in patients belonging to the high-risk subgroup based on the survival analysis. Further, the Cox regression analysis indicates risk scores have the ability to predict the prognosis of patients with melanoma independently. In addition, The prognosis prediction model using the four m^7^GMRRGs had better predictive power when combined with the clinical factors. The above results suggested that *EIF4E3, LARP1, NCBP3,* and *IFIT5* are promising prognostic markers for melanoma. Studies show *EIF4E3* is a subtype of the eukaryotic translation initiation factor *EIF4E,* which competes with the pro-growth function of *EIF4E* by regulating the cap-binding activity of eukaryotic translation, thereby inhibiting the oncogenic transformation and cell proliferation^44^. Previous studies reveal a decrease in *EIF4E3* expression increases tumor cell activity and aggressiveness in the head, and neck squamous cell carcinomas, acute myeloid leukemia, and breast cancer^45–47^. *LARP1* has recently been identified as an oncogenic RNA binding protein, and studies suggest the involvement of *LARP1* in promoting cell proliferation and invasion in lung cancers^48^ and colorectal cancers^49^. Moreover, *LARP1* expression levels were considerably elevated in ovarian cancer and were correlated with poor clinicopathological characteristics in patients with ovarian cancer^50^. *NCBP3* enhances the aggressiveness of glioma by inhibiting *GBX2* transcription in glioma^51^. *IFIT5* belongs to the interferon-inducible tetrapeptide repeat protein (IFIT) family. It is an essential component of the antiviral immune response^52^ and acts as a tumor suppressor by participating in the apoptotic pathway^53^. The current research describes for the first time that the risk scores created by *EIF4E3*, *LARP1*, *NCBP3*, and *IFIT5* can serve as independent prognostic factors for melanoma.

A recent research has highlighted that the immune cells in the tumor microenvironment are essentially involved in tumorigenesis and development^54^. Genetic polymorphisms in melanoma lead to the formation of many neoantigens. Melanoma is highly immunogenic and can trigger specific anti-cancer immune responses. Hence immunotherapy could be an excellent candidate for treating melanoma^55^. Our analysis reveals lower immune cell infiltration in the high-risk subgroup, compared with that in the low-risk subgroup. A previous study suggested that CD8^+^ T cells are key components of the intrinsic immune response and positively correlate with Treg cell recruitment^56^ In contrast, the immune escape mechanism of tumors is associated with CD8^+^ T cell depletion^57^. The number of CD8^+^ T cells could be used as a predictor of prognosis in cancer^58^. Based on the current results and existing literature, it could be assumed that m^7^GMRRGs may affect the prognosis of patients with melanoma by modulating the immune response.

In recent years, targeted tumor immunotherapy has been successful in the treatment of aggressive malignancies^59^. Targeted tumor antigens (TAs) are key to developing safe and effective anti-cancer immunotherapy. However, dysregulation of RNA methylation may affect anti-cancer immunotherapy^60^. Partial RNA methylation modification inhibitors which may be a breakthrough for novel immunotherapy strategies have effectively controlled tumor progression^61^. Thus, checkpoint inhibitor-based immunotherapy may be keys to future melanoma treatment. We found significant variations in the expression of multiple immune checkpoints, such as CD70, TIGIT, PDCD1 (PD-1), LAG3, and LGALS9, between the two risk subgroups, thereby indicating that m^7^GMRRGs may have the potential to be used as an immunotherapeutic target. In addition, drug sensitivity analysis revealed patients in high-risk subgroup were highly sensitive to the common chemotherapeutic agents like lapatinib, gefitinib, dasatinib, cisplatin, bosutinib, and bortezomib.

Results of our study indicate a association between m^7^GMRRGs and melanoma prognosis, and the prognostic prediction model using m^7^GMRRGs may predict the prognosis of patients with melanoma well. Nevertheless, these results may provide a clue for potential better options of melanoma treatment but need further validation in futural studies.

## Supplementary Information

Supplementary Legends.

Supplementary Figure 1.

## Data Availability

The original contributions to this research are included in the article/supplementary material. Further queries can be directed to the corresponding author.
