# Supplementary material for: N7-methylguanosine methylation-related regulator genes as biological markers in predicting prognosis for melanoma
Source: Sci Rep. 2022 Dec 6;12:21082. doi: 10.1038/s41598-022-25698-x (PMC9726938; doi:10.1038/s41598-022-25698-x)
Supplement: Supplementary file 1 — Supplementary Legends. [file 41598_2022_25698_MOESM1_ESM.docx]

**Supplemental Figure 1** Association between risk scores and clinical features of patients with melanoma (*P* > 0.05).

**Abbreviations:** T, tumor stage; N, lymph node; M, distant metastasis
