# Supplementary figures and images for: N7-methylguanosine methylation-related regulator genes as biological markers in predicting prognosis for melanoma
Source: Sci Rep. 2022 Dec 6;12:21082. doi: 10.1038/s41598-022-25698-x (PMC9726938; doi:10.1038/s41598-022-25698-x)

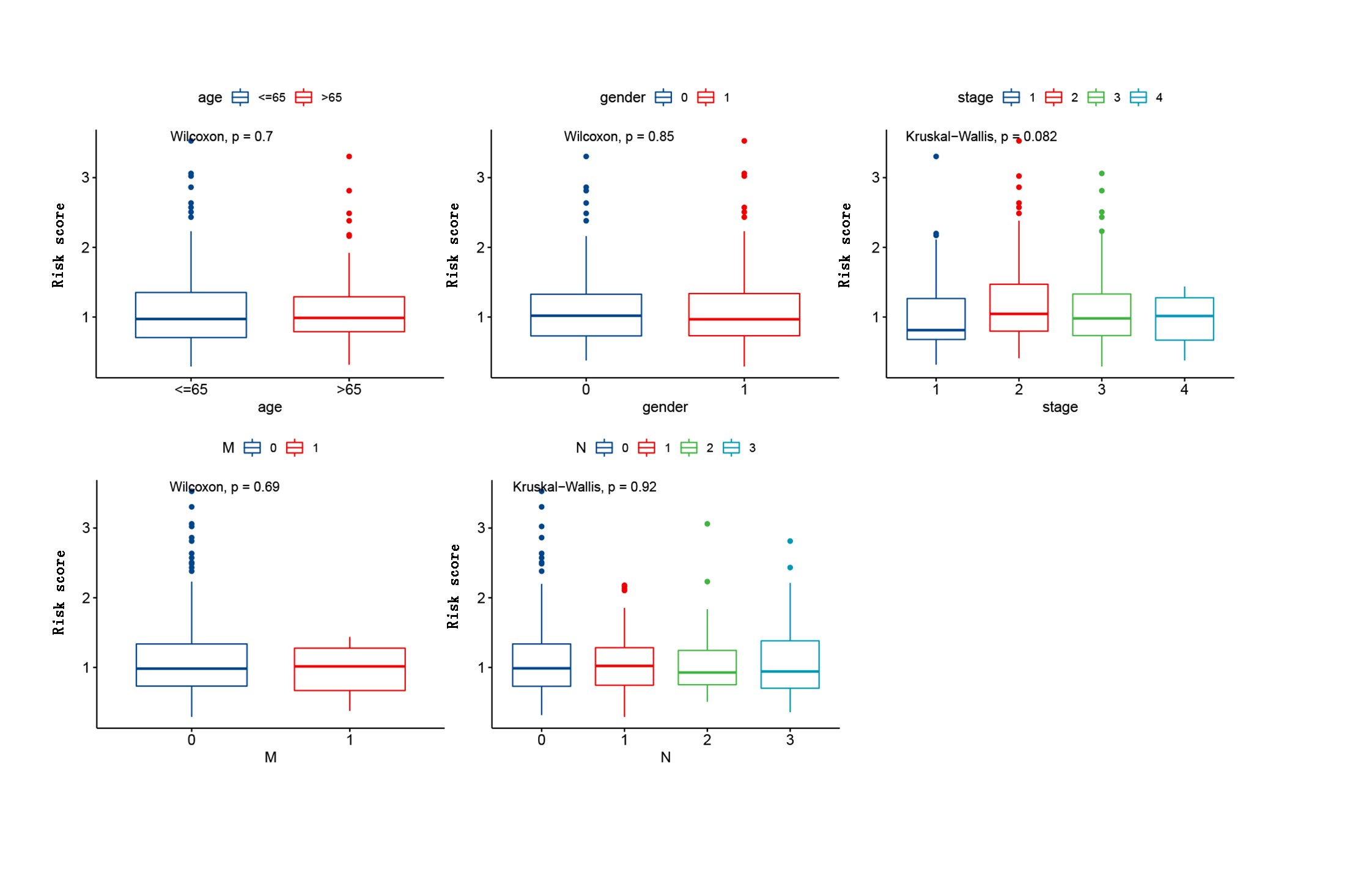

Supplement: Supplementary file 2 — Supplementary Figure 1. [file 41598_2022_25698_MOESM2_ESM.tif]
